# Comprehensive resistome and antibiotic profiling in wastewater and biosolids: a case study

**DOI:** 10.3389/fmicb.2026.1830828

**Published:** 2026-06-22

**Authors:** Alba Lara-Moreno, Fernando Madrid, Marina Rubio-Bellido, Chiara Borsetto, William Nurmi, Esteban Alonso, Juan L. Santos, Jaime Villaverde

**Affiliations:** 1 Department of Microbiology and Parasitology, Faculty of Pharmacy, University of Seville, Seville, Spain; 2 Department of Soils and Water, Institute of Natural Resources and Agrobiology of Seville, Spanish National Research Council (IRNAS-CSIC), Seville, Spain; 3 Resistomap Oy, Helsinki, Finland; 4 Department of Analytical Chemistry, University of Seville, Seville, Spain

**Keywords:** antibiotic resistance gene index, antibiotic resistance genes, antimicrobial resistance, environmental dissemination, mobile genetic elements, sewage sludge composting, wastewater treatment plants

## Abstract

Wastewater treatment plants (WWTPs) are widely recognized as hotspots for the accumulation of antimicrobial resistance determinants (AMR). This study analyses the presence of antibiotic resistance genes (ARGs), mobile genetic elements (MGEs), and antibiotic residues in influent and effluent wastewater, as well as in dehydrated and composted sludge from a municipal WWTP including a composting facility in southern Spain. Influent wastewater exhibited different ARG profiles, including macrolide–lincosamide–streptogramin (MLSB), aminoglycoside, tetracycline, quinolone, and multidrug resistance related genes, alongside MGEs such as *intI1*, *IS26*, and *qepA*. Although treatment processes reduced microbial loads and the relative abundance of ARGs, several ARGs and MGEs persisted in effluent water. Dehydrated Sludge matrices showed the presence of tetracycline and macrolide antibiotics (e.g., doxycycline and azithromycin), consistent with their physicochemical properties related with their environmental persistence. Composting treatment led to an effective decreasing in the global ARG load, however, key MGEs, including integrons, insertion sequences, and transposons, remained detectable, indicating the persistence of residual genetic reservoirs, which can be justified by the presence of antibiotics at low concentration and/or co-occurring stressors. The application of resistance metrics, such as the Antibiotic Resistance Gene Index (ARGI) and the Standard Resistance Gene Index (SRGI), enabled comparison with European benchmark data, revealing that ARG loads detected in wastewater and sludge matrices were consistent with previously reported regional data. The detection of clinically relevant genes (e.g., *mcr-1*, *qacEΔ1*, and *merA*) indicates that these matrices may act as reservoirs of resistance determinants of public health concern. Overall, these findings highlight the value of molecular surveillance and resistance indices and the need for longer-term studies of AMR in wastewater and sludge systems.

## 1 Introduction

Antimicrobial resistance (AMR) is widely recognized as one of the most pressing public health challenges of the 21st century. Within the European Union, multidrug-resistant bacteria are estimated to cause approximately 33,000 deaths annually (European Action Plan “One Health,” COM/2017/0339 final), and recent global projections indicate that AMR-associated mortality may increase substantially over the coming decades without effective mitigation strategies (Kariuki, 2024).

Beyond its impact on morbidity and mortality, AMR is also expected to generate profound economic and societal consequences, including significant losses in global productivity and increased vulnerability of human populations (United Nations Environment Programme (UNEP), 2023). Together, these dimensions reinforce the need for coordinated actions within the One Health framework, including human, environmental, and agricultural perspectives (European Commission, 2017).

Central to this crisis is the environmental dissemination of antibiotic resistance genes (ARGs), often facilitated by mobile genetic elements (MGEs) such as plasmids, transposons, and integrons, which enable horizontal gene transfer (HGT) across diverse microbial communities (Berendonk et al., 2015). Wastewater treatment plants (WWTPs) have been identified as key nodes for the convergence of antibiotics, antibiotic-resistant bacteria (ARB), ARGs, and MGEs originating from domestic, hospital, agricultural, and industrial sources (Wei et al., 2024).

Although WWTPs effectively reduce overall pathogen loads, they are not specifically designed to remove genetic determinants of resistance, and ARGs and MGEs may persist throughout treatment processes (Gillings et al., 2015) and be discharged into receiving environments via treated effluents (One Health Trust, 2018). During treatment, changes in microbial community composition and exposure to residual selective agents may influence ARG profiles, although such effects are difficult to disentangle in full-scale operational systems. As a result, treated effluents frequently contain detectable ARGs, raising concerns regarding their environmental persistence (One Health Trust, 2018).

Sewage sludge, the semi-solid byproduct of wastewater treatment, is particularly enriched in microbial biomass and associated genetic material. Numerous studies have reported high diversity and abundance of ARGs in sludge, including genes conferring resistance to tetracyclines, sulfonamides, macrolides, and *β*-lactams (Wei et al., 2024). The presence of MGEs in sludge provides genetic contexts that may contribute to the long-term maintenance of resistance determinants. The land application of treated sludge or biosolids therefore represents a potential pathway for the introduction of ARGs and MGEs into soil ecosystems, where they may persist within indigenous microbial communities (Qin et al., 2022).

The role of sewage sludge composting in ARG attenuation remains an active area of investigation. While thermophilic composting can reduce overall microbial abundance, its effectiveness in mitigating ARGs and MGEs remains variable and gene-specific. Previous studies have shown that composting can reduce the abundance of several sulfonamide and tetracycline resistance genes, while exhibiting limited effects on macrolide resistance genes and integrons (Fu et al., 2021). In addition, microbial succession during composting, particularly during maturation phases, may favor the persistence of specific resistant taxa (Zhang X. et al., 2025), highlighting ongoing uncertainty regarding the fate of resistance determinants in composted sludge.

The environmental occurrence of antibiotics themselves is an additional factor shaping resistance profiles in wastewater and sludge systems. Antibiotics are frequently detected in influent and effluent wastewater, as well as in sludge and composted materials, due to incomplete removal and compound-specific physicochemical properties. Experimental studies have shown that even sub-inhibitory concentrations of antibiotics can promote the maintenance and horizontal transfer of ARGs (Berendonk et al., 2015; Bengtsson-Palme and Larsson, 2016).

Despite the extensive body of literature addressing ARGs in wastewater and sludge, assessments that simultaneously examine antibiotics, ARGs, MGEs, and quantitative resistance indices across multiple treatment matrices within a single full-scale WWTP remain comparatively limited, especially in southern European contexts. Furthermore, comparative tools such as the Antibiotic Resistance Gene Index (ARGI) and the Standard Resistance Gene Index (SRGI), which allow normalization of ARG abundance and contextualization against regional reference datasets, remain underutilized in snapshot-type environmental studies.

In this context, the present study provides a site-specific assessment of ARGs, MGEs, and selected antibiotic residues across influent wastewater, effluent wastewater, dehydrated sludge, and composted sludge from a municipal WWTP located in Seville (southern Spain).

The specific objectives of this study were: (i) to characterize the occurrence and relative abundance of ARGs and MGEs across wastewater and sludge treatment stages; (ii) to quantify selected antibiotic residues in the same matrices; and (iii) to contextualize resistance profiles using indices (ARGI and SRGI) relative to available European benchmarks. This study seeks to contribute comparable system-level data to ongoing One Health initiatives focused on environmental reservoirs of AMR.

## 2 Materials and methods

### 2.1 Study site

In this study, four types of matrices were collected: dehydrated (non-composted) sludge, composted sludge, influent wastewater, and treated effluent wastewater. All samples were obtained from the Tablada Wastewater Treatment Plant (EDAR Tablada) and from the Advanced Composting Plant at the Copero Environmental Complex. EDAR Tablada, located on Ctra. de la Esclusa, km 2 in the western area of Seville, has been in operation since 1990 and has a treatment capacity of 50,000 m^3^ day^−1^, serving a design population equivalent of 200,000 inhabitants. It receives wastewater from the western districts of Seville as well as from some surrounding municipalities. The treatment line comprises physical pre-treatment (screening and grit removal), primary sedimentation, a medium-load activated-sludge biological process, and anaerobic digestion of the generated sludge. Dehydrated sludge was collected after the last stage before composting at the advanced composting plant, El Copero, which processes approximately 70,000 t of sludge annually through controlled aerated composting.

#### 2.1.1 Sampling strategy and collection

Wastewater and sludge samples were collected from a WWTP and a connected composting facility. Four different samples were collected: influent wastewater, treated effluent wastewater, dehydrated sludge, and composted sludge. Wastewater samples were 24-h composite samples (10 L) collected from the inlet and outlet of the WWTP. Sludge samples were collected at different stages in the composting facility, before stabilization (dehydrated sludge) and after composting (composted sludge). This approach was designed to study the co-occurrence of antibiotics, ARGs, and MGEs across all key treatment stages in a full-scale system.

#### 2.1.2 Sewage sludge matrices characterization

Dehydrated and composted sewage sludge samples provided by EMASESA were characterized using standardized analytical methods. Dry matter content was determined by gravimetric method and afterwards oven drying at 105 °C until constant weight (ISO 11465). Total organic matter was quantified by loss on ignition at 550 °C (ISO 10694). Sample pH was measured potentiometrically in a 1:5 (w/v) sludge–water suspension (ISO 10390).

Total carbon and nitrogen contents were determined using elemental analysis based on dry combustion (ISO 10694 for carbon and ISO 13878 for nitrogen), and the C/N ratio was calculated accordingly. Total nitrogen was determined using the Kjeldahl digestion method (ISO 11261), and ammoniacal nitrogen was measured colorimetrically after aqueous extraction following APHA standard method 4,500-NH₃.

Macronutrients, including phosphorus, potassium, calcium, and magnesium, were quantified after acid digestion of dried samples using a nitric–perchloric acid mixture (ISO 11885). Elemental concentrations were determined by inductively coupled plasma optical emission spectrometry (ICP-OES). All analyses were performed in triplicate, and results were expressed on a dry weight basis.

Heavy metal concentrations in sewage sludge were determined using Inductively Coupled Plasma Mass Spectrometry (ICP-MS). Prior to instrumental analysis, sludge samples were subjected to microwave-assisted acid digestion using concentrated nitric acid, and when required hydrochloric acid, to ensure complete solubilization of metal species and accurate quantification.

The detection of *Salmonella* spp. in sewage sludge was performed in accordance with ISO 6579-1. Two selective enrichments of the sludge samples were performed in Buffered Peptone Water (BPW) and growth in Rappaport-Vassiliadis Soya (RVS) broth and Mueller-Kauffmann Tetrathionate-Novobiocin (MKTTn) broth. Isolation was subsequently carried out on selective chromogenic agar for Salmonella. Quantification of *Escherichia coli* was carried out according to ISO 16649-2 standard, using plating serial dilutions on TBX (Tryptone Bile X-glucuronide) agar. Results were expressed as colony-forming units per gram of sludge (CFU g^−1^).

### 2.2 High-throughput quantitative PCR. Resistome analysis

Resistome analysis have been conducted in the sludge and wastewater DNA samples. DNA was extracted from sludge samples using the Qiagen DNeasy PowerSoil Pro Kit (Qiagen, Germany) and from wastewater samples using the DNeasy PowerWater Kit, according to the manufacturers’ instructions. The extracted DNA was quantified using NanoDrop One (Thermo Fisher Scientific). A panel of 384 genes with a 304-ARG subset plus MGEs/pathogen or taxonomic markers and 16S rRNA (also referred hereafter as ARG 2.1 panel) was selected and analyzed using Smartchip qPCR at Resistomap Oy (Zhu et al., 2013; Muziasari et al., 2016; Stedtfeld et al., 2018). The overall bacterial load was determined by measuring the 16S rRNA gene in each sample in order to normalize the other detected genes and calculate their relative abundance using the ΔCt method, using the mean Ct value of three technical replicates. Each reaction volume of 100 nL consisted of 1X Smartchip TB Green Gene Expression Master Mix following manufacturer’s instructions, 20 nL of DNA template, 300 nM of forward and reverse primers, and nuclease-free water. For the Smartchip qPCR run, an initial denaturation step at 95 °C for 10 min was performed, followed by 40 cycles of denaturation at 95 °C for 15 s, annealing at 60 °C for 15 s, and extension at 72 °C for 30 s. Melting curve analysis was measured at the end with an increase to 97 °C by 0.4 °C/step. Raw data were processed to exclude primer dimers using melting-curve analysis and the threshold cycle (Ct) of 27 was used as a cut-off limit for detection (Muziasari et al., 2016; Stedtfeld et al., 2018). Replicate Ct standard deviation and efficiency were also considered in the data filtration process. For each sample tested, only reactions with three or two technical replicates were used for the calculation of the mean Ct for downstream analysis. ARG quantification results were expressed per milliliter of influent and effluent wastewater samples and per gram of dry weight for sludge samples.

### 2.3 ARG index and standard resistance gene index computation

Antibiotic Resistance Gene Index (ARGI) represents the overall ARG load in the measured bacterial community. ARGI was calculated individually for each sample according to Resistomap’s methodology (Silvester et al., 2025), but with a custom set of the studied 304 ARGs included in the Resistomap’s ARG2.1 panel used in this study, as Equation 1:


ARGI=5+mean[log10(ra)] (1)

where ra is the relative abundance. Values below 0 are set to 0; the index is capped at 5.

ARGI is useful as a measure of antibiotic resistance load in environmental matrices for comparing bacterial communities between this research and one another and, more broadly, with samples that have measured the same set of ARGs. Therefore, this study have also included the Standardized Resistance Gene Index (SRGI), which is calculated based on a standardized set of 62 ARGs selected by Resistomap for clinical and environmental relevance. Neither ARGI nor SRGI is well-suited for measuring treatment efficiency, since it is calculated as a proportion of bacterial abundance and thus does not capture the overall reduction in bacteria.

For references, European mean ARGIs were recalculated from Resistomap’s database for the studied ARG 2.1 panel. Resistomap’s commercial database includes ~402 influent wastewater, 517 treated effluent wastewater and 685 sludge samples from multiple European countries and additional global datasets in Africa, Asia and North America, all analyzed by a standardized and robust qPCR process (Lai et al., 2021; Elbait et al., 2024; Robins et al., 2024). Continental reference ARGIs were calculated using the same assays as for the samples in this study, with missing assays estimated from the measured assays, ensuring each assay contributed once per sample.

### 2.4 Antibiotic chemical analysis

Analytical determination was performed in an Agilent 1,290 Infinity II chromatograph (Agilent, USA) equipped with a vacuum degasser, a binary pump, and an automatic injector and coupled to an Agilent 6,495 triple quadrupole (QqQ) mass spectrometer equipped with an electrospray ionization source (Agilent Technologies, Santa Clara, CA, USA).

#### 2.4.1 Sludge samples

##### 2.4.1.1 Tetracyclines

Tetracyclines were analysed in sludge samples according to García-Criado et al. (2025). Briefly, tetracyclines were extracted from 0.1 g of freeze-dried sludge using matrix-solid phase extraction (MSPD) with PSA and Florisil as clean-up sorbents and silica as dispersant. Antibiotics were eluted from the cartridge by adding three consecutive 4 mL aliquots of a mixture of acetone and Na_2_EDTA-McIlvaine buffer (pH 4) (30:70, *v/v*). The eluates were evaporated and the remaining aqueous extract was transferred to a 10-mL volumetric flask and diluted to the mark with pure water, filtered through a 0.22 μm regenerated cellulose syringe filter and collected in an automatic injector vial for online SPE-LC–MS/MS determination.

A 100-μL aliquot of the extract was transferred to a reversed-phase Bond Elut Online PLRP-S cartridge by passing a water solution containing 2 mM ammonium formate. An ammonia solution was used to adjust pH to 7.0. After sample loading, the retained compounds were transferred onto the analytical column by the flow of the mobile phase. For analytes separation a Zorbax RRHD Eclipse Plus C18 column (150 mm × 3.0 mm i.d., 1.8 μm particle size, Agilent Technologies, Santa Clara, CA, USA) thermostated at 35 °C was used. The column was protected with a Zorbax RRHD Eclipse Plus C18 guard column (3.0 mm i.d., 1.8 μm particle size, Agilent Technologies, Santa Clara, CA, USA). A 10 mM ammonium formate buffer was employed as the mobile phase, containing formic acid (0.1%, *v/v*) and 0.5 mM ammonium fluoride (solvent A) and methanol (solvent B). Analytes were eluted by gradient elution at a flow rate of 0.3 mL min^−1^ in a total run time of 25 min. LC–MS/MS parameters for each compound are provided in Table 1 (García-Criado et al., 2025). For each batch of samples, blanks, spiked samples, and matrix-matched calibration curves were prepared and analyzed as quality control. Analytical validation parameters can be found in García-Criado et al. (2025).

**Table 1:** LC–MS/MS parameters for the determination of the antibiotics.

Antibiotic	Precursor ion *(m/z)*	Product ions (quantifier/qualifier) *(m/z)*	CE (eV)	Ion ratio
Macrolides				
Roxithromycin	838.1	158.1/679.4	32/20	72.9
Azithromycin	750.0	591.5/116.1	28/44	56.1
Erythromycin	734.5	83.0/576.4	68/20	81.4
Clarithromycin	749.0	158.1/590.4	28/16	49.5
Fluoroquinolones				
Norfloxacin	320.3	302.1/231.0	24/44	26.0
Enrofloxacin	360.4	286.1/342.1	40/40	61.7
Ciprofloxacin	332.1	314.1/231.0	16/40	98.5
Flumequine	262.3	244.0/202.0	20/36	60.5
Nadifloxacin	361.4	343.1/283.0	28/44	97.6
Lomefloxacin	352.4	265.1/334.1	20/24	95.6
Ofloxacin	362.2	318.2/261.2	20/28	74.2
Tetracyclines				
Tetracycline	445.2	410.2/154.1	20/30	57.9
Doxycycline	445.2	428.0/154.0	30/30	41.8
Chlortetracycline	479.1	444.0/462.0	20/20	64.0
Minocycline	458.2	441.1/283.1	20/48	22.4
Tigecycline	586.3	513.3	43	–
Metacycline	443.0	426.0/381.0	25/25	31.6
Diaminopyridines				
Trimethoprim	291.2	261.1/229.8	28/24	98.2
Sulfonamides				
Sulfamethoxazole	254.3	92.1/108.0	28/28	76.1
Sulfadiazine	251.3	92.1/156.0	28/12	98.0
Sulfamethazine	279.3	186.0/92.0	16/36	76.4
*β*-lactams				
Ampicillin	350.1	160.1/114.0	12/80	3.72
Amoxicillin	366.1	114.0/349.1	14/4	95.8
Penicillin V	351.1	160.1/114.1	13/33	40.2
Penicillin G	335.1	176.1/160.1	12/12	74.7

CE: collision energy.

##### 2.4.1.2 Other antibiotics

The rest of the antibiotics were analyzed in sludge samples according to Mejías et al. (2023). Briefly, antibiotics were extracted from 1 g of lyophilized sludge using ultrasound-assisted extraction (UAE) with three successive 3 mL aliquots of methanol 0.5% (*v/v*) in an ultrasonic bath for 15 min and the obtained extract was cleaned up with 0.4 g of C18 sorbent. The liquid phase was transferred to another tube and evaporated to dryness under a gentle nitrogen stream. The dried extract was dissolved in 0.3 mL of methanol:water solution (1:1, *v/v*), filtered through a 0.22 μm cellulose syringe filter and collected in an automatic injector vial for LC–MS/MS determination.

Chromatographic separation was carried out in a Zorbax RRHD Eclipse Plus C18 (150 mm × 3.0 mm i.d., 1.8 μm particle size) column (Agilent, USA), protected with a Zorbax RRHD Eclipse Plus C18 (3.0 mm i.d., 1.8 μm particle size) guard column (Agilent, USA) and thermostated at 35 °C. Antibiotics were eluted from the column by gradient elution with a flow rate of 0.4 mL min − 1 using a mobile phase composed of 10 mM ammonium formate (0.05% v/v, formic acid) and methanol. Injection volume was 10 μL. LC–MS/MS parameters for each compound are given in Table 1. For each batch of samples, blanks were prepared, spiked samples. Matrix-matched calibration curves were processed as quality control. Accuracy, precision and MQL parameters are described in Mejías et al. (2023).

#### 2.4.2 Wastewater samples

##### 2.4.2.1 Tetracyclines

Tetracyclines were analyzed in wastewater samples by online SPE-LC–MS/MS. A 900-μL aliquot of the wastewater filtered sample (0.22 μm regenerated cellulose syringe filter) was mixed with 100 μL of Na_2_EDTA-McIlvaine buffer (pH 4) in an automatic injector vial. A 100-μL aliquot of the sample was loaded into the online SPE loop as it was described in section 1.1. For each batch of samples, procedural blanks, standard addition calibration from 5 ng L^−1^ to 10 μg L^−1^ and spiked samples were processed as quality control. Method determination limits (MDL) and MQL can be found in Table 2.

**Table 2:** Method limits of quantification and detection in influent and effluent wastewater.

Compound	Method limit of quantification (ng L^−1^)		Method limit of detection (ng L^−1^)	
	Influent	Effluent	Influent	Effluent
Sulfadiazine	23.9	15.2	12.2	9.54
Sulfamethoxazole	7.62	7.32	1.52	1.46
Sulfamethazine	3.91	3.84	1.57	1.54
Erythromycin	0.44	0.08	0.13	0.03
Azithromycin	10.2	2.18	6.81	1.45
Clarithromycin	4.50	0.50	1.79	0.20
Roxithromycin	15.0	12.0	10.5	9.52
Trimethoprim	3.59	2.02	1.44	0.81
Ciprofloxacin	1.29	0.20	0.42	0.15
Ofloxacin	1.30	0.50	0.40	0.15
Enrofloxacin	0.35	0.25	0.10	0.07
Flumequine	0.41	0.39	0.13	0.12
Norfloxacin	0.50	0.35	0.15	0.10
Nadifloxacin	0.37	0.35	0.12	0.11
Lomefloxacin	797	249	159	49.8
Amoxicillin	1874	1,674	943	735
Ampicillin	48.9	47.1	24.4	23.6
Penicillin G	1943	1754	1,028	762
Penicillin V	35.6	24.8	17.9	12.4
Tetracycline	0.10	0.05	0.06	0.03
Doxycycline	0.27	0.08	0.08	0.03
Oxytetracycline	0.19	0.08	0.06	0.03
Chlortetracycline	0.72	0.25	0.22	0.08
Minocycline	0.55	0.44	0.17	0.13
Tigecycline	0.52	0.35	0.16	0.11
Metacycline	0.55	0.27	0.17	0.08

##### 2.4.2.2 Other antibiotics

The rest of the antibiotics were analysed in wastewater samples by online SPE-LC–MS/MS. A 600-μL aliquot of the wastewater filtered sample (0.22 μm regenerated cellulose syringe filter) was loaded into the online SPE loop. Then, the extract was transferred to a reversed-phase Bond Elut Online PLRP-S cartridge by passing water at a flow rate of 2 mL min^−1^ for 2 min. After sample loading, the switching valve was moved to the elution position to transfer retained compounds onto the analytical column by the flow of the mobile phase. A flow rate of 2.0 mL min^−1^ for 4.4 min SPE cartridge was washed with methanol and afterwards conditioned with water for 3.4 min at a flow rate of 2.0 mL min^−1^. Chromatographic separation was carried out in a Zorbax RRHD Eclipse Plus C18 (150 mm × 3.0 mm i.d., 1.8 μm particle size) column (Agilent, USA), thermostated at 35 °C and protected with a Zorbax RRHD Eclipse Plus C18 (3.0 mm i.d., 1.8 μm particle size) guard column (Agilent, USA). Antibiotics were eluted from the column by gradient elution with a flow rate of 0.4 mL min^−1^ using a mobile phase composed of 10 mM ammonium formate (0.05% *v/v*, formic acid) and methanol. Detailed LC–MS/MS parameters for each compound are provided in Supplementary Table 1). For each batch of samples, standard addition calibration from 5 ng L^−1^ to 5 μg L^−1^ and spiked samples served as quality control. Method determination limits (MDL) and MQL can be found in Supplementary Table 2.

## 3 Results

### 3.1 Antibiotic-resistant genes abundance

**Figure 1:**
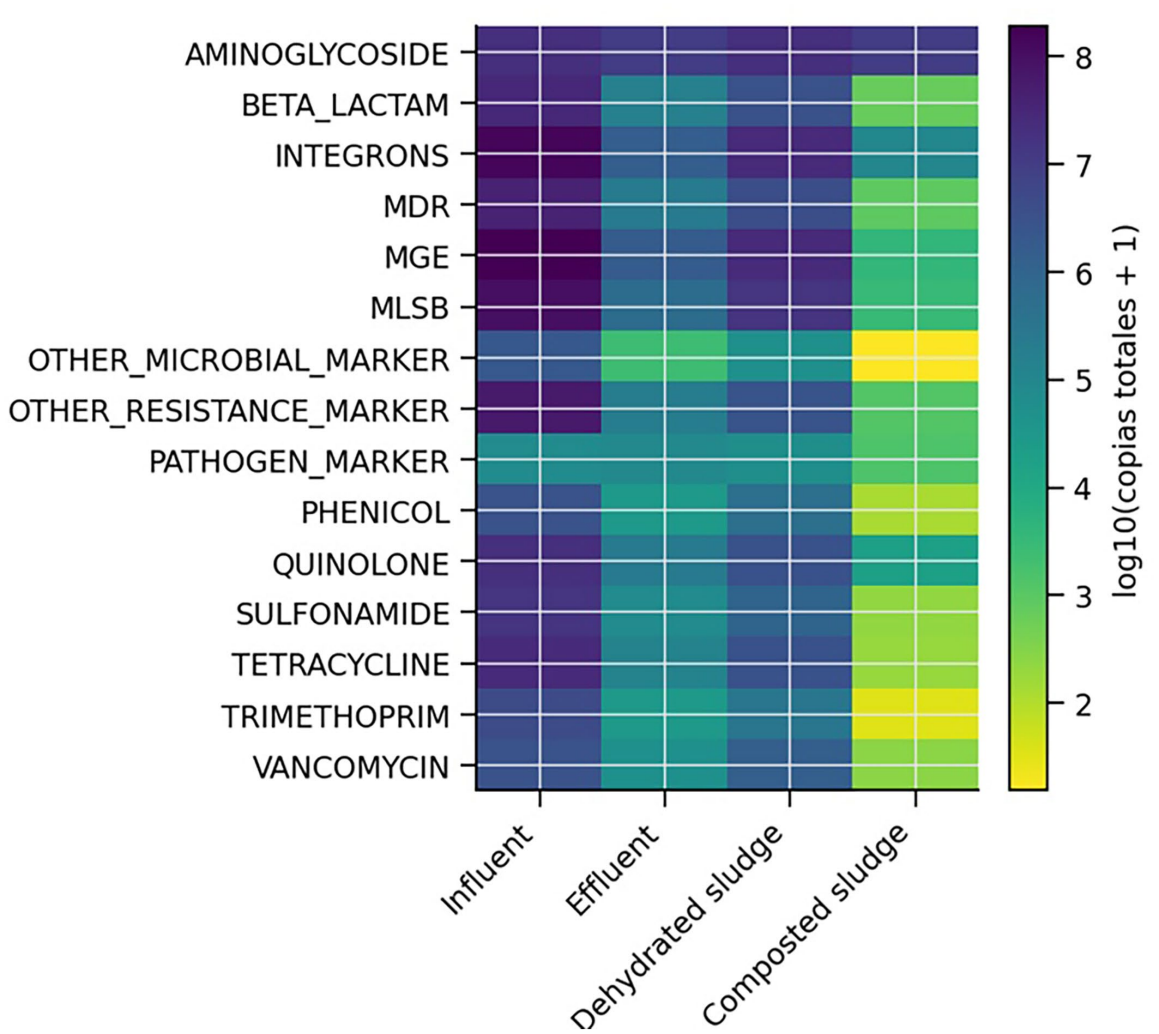
Heatmap showing total gene copy numbers aggregated by gene group for each matrix. Values represent the sum of all genes within each group and were log10-transformed (copies +1). Darker colors indicate higher total gene abundance.

Relative abundance measurements of ARGs, taxonomic pathogen markers, and MGEs were analysed in the four investigated samples (Figure 1): dehydrated sludge, composted sludge, influent wastewater, and effluent wastewater. The results showed different resistance determinants profiles for the different studied matrices.

The wastewater samples showed significant concentrations of ARGs and MGEs, especially in influent wastewater. Among the ARG groups, macrolide-lincosamide-streptogramin (MLSB) genes reached 9.7 × 10^7^ copies/mL, aminoglycoside resistance genes 21.3 × 10^6^ copies/mL, and tetracycline resistance genes 2.7 × 10^7^ copies/mL. Quinolone resistance genes were also prominent, with a total of 2 × 10^7^ copies/mL. Beta-lactam resistance genes accounted for 2.7 × 10^7^ copies/mL, while sulfonamide and trimethoprim resistance genes contributed 1.5 × 10^7^ and 4.4 × 10^6^ copies/mL, respectively. Phenicol resistance genes were present at 2.8 × 10^6^ copies/mL, and vancomycin resistance genes at 2.8 × 10^6^ copies/mL. Multidrug resistance genes totalled 3.9 × 10^7^ copies/mL.

MGEs were particularly abundant, with a cumulative concentration of 1.9 × 10^8^ copies/mL in influent water. Specific genes such as *intI1* (77.6 × 10^6^ copies/mL), *sul1_2* (11.6 × 10^6^ copies/mL), *blaOXA48* (1.9 × 10^5^ copies/mL), *qepA* (12.1 × 10^6^ copies/mL), and *IS26* (33.5 × 10^6^ copies/mL) were among the most prevalent. After treatment, these genes remained detectable in effluent water, with *intI1* at 9.9 × 10^5^ copies/mL, *sul1_2* at 5.3 × 10^4^ copies/mL, *blaOXA48* at 3.6 × 10^3^ copies/mL, *qepA* at 1.9 × 10^5^ copies/mL, and *IS26* at 76,700 copies/mL. The total concentration of MGEs in effluent water was approximately 1.6 × 10^6^ copies/mL.

The comparative analysis of gene copy numbers in sludge and composted sludge revealed substantial differences in the abundance of antibiotic resistance determinants and associated MGEs. In dehydrated sludge, the most abundant gene groups included other taxonomic pathogen markers (7.3 × 10^3^ copies/g), integrons (25.7 × 10^6^ copies/g), MGE (27.3 × 10^6^ copies/g), MLSB (16.4 × 10^6^ copies/g), and aminoglycoside (1 × 10^7^ copies/g). After composting, these totals were drastically reduced, with integrons dropping to ~9.8 × 10^4^ copies/g, MGE to ~3.9 × 10^3^ copies/g, and MLSB to ~3.1 × 10^3^ copies/g.

Among individual genes, several showed notable persistence despite composting. The integron marker *intI1_1* decreased from ~18.4 × 10^6^ to ~9.7 × 10^4^ copies/g, remaining the most abundant integron gene post-treatment. The quinolone resistance gene *qepA* decreased from ~2.34 × 10^6^ to ~1.8 × 10^4^ copies/g, while the macrolide resistance gene *mphA* from ~2.5 × 10^6^ to ~639 copies/g. The sulfonamide resistance gene *sul1_2*, often associated with class 1 integrons, declined from ~2.7 × 10^5^ to ~182 copies/g.

The aminoglycoside resistance gene *aadA7* remained detectable at 3.6 × 10^6^ copies/g in the composted sample, increasing from 2.1 × 10^6^. The tetracycline resistance gene *tet*(L)*_2* was reduced from 1.9 × 10^5^ to 88 copies/g. In contrast, the beta-lactamase gene *blaOXA48*, which confers resistance to carbapenems, dropped from 6.9 × 10^4^ copies/g in dehydrated sludge to undetectable levels. The mobile element *IS1111*, known for its role in ARG mobilization, also became undetectable after composting, despite an initial abundance of 8.6 × 10^5^ copies/g in dehydrated sludge.

### 3.2 Presence of antibiotics

**Figure 2:**
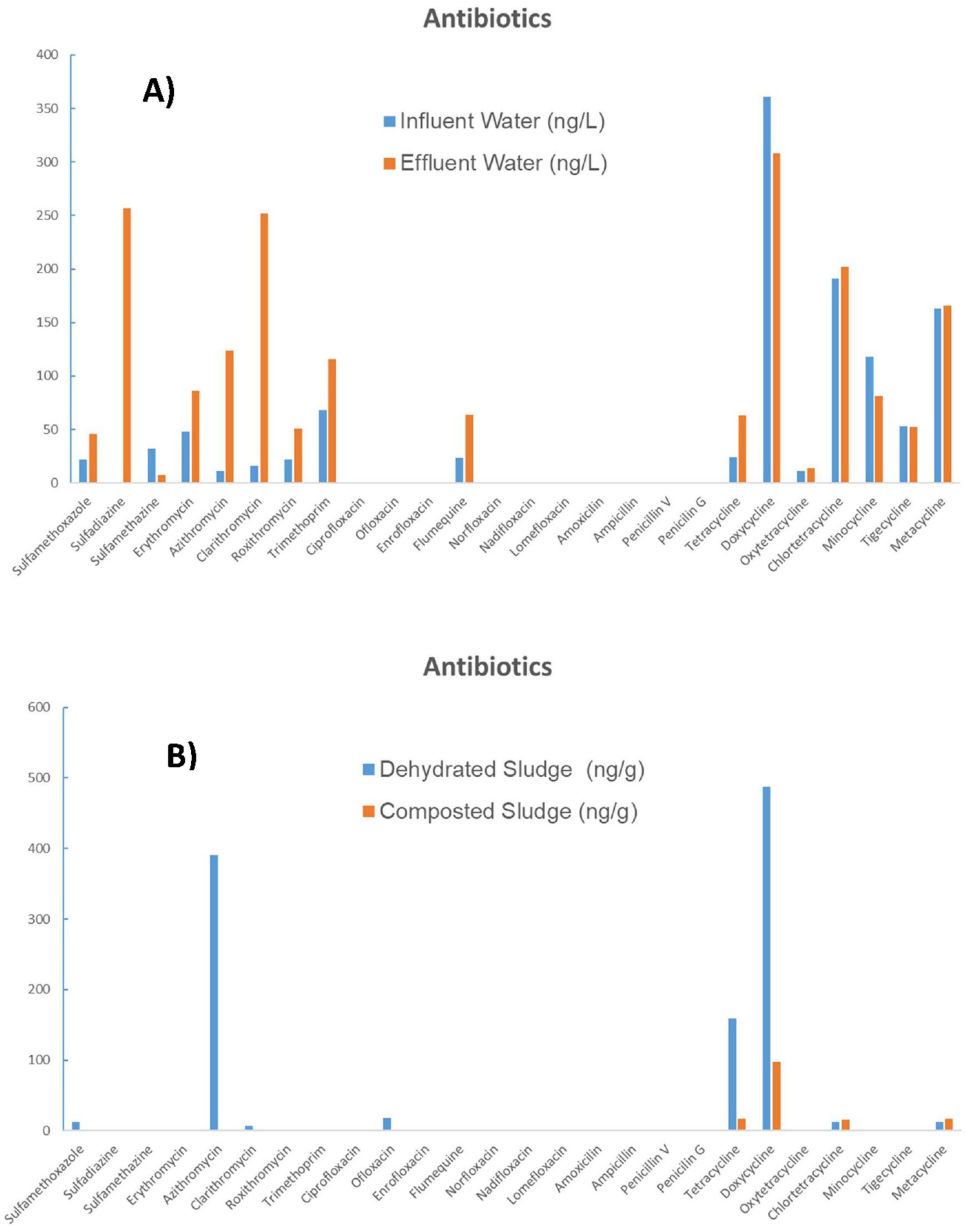
Presence of persistent antibiotics in influent and effluent wastewater, dehydrated sludge, and composted sludge, and classified according to clinical criticality. Antibiotics are categorized based on their importance for human medicine as **(A)** low-criticality (important antibiotics with multiple therapeutic alternatives); **(B)** medium-criticality (highly important antibiotics used for serious infections with limited alternatives); **(C)** high-criticality antibiotics (critically important or “last-resort” compounds reserved for the treatment of severe or multidrug-resistant infections).

Tetracyclines dominated the detected antibiotic profiles (Figure 2), with doxycycline showing the highest concentrations in influent (361 ng/L), effluent (308 ng/L), and dehydrated sludge (487 ng/g). Similarly, tetracycline (159 ng/g) and chlortetracycline (13–16 ng/g) were prominent in sludge, while oxytetracycline was detected at lower levels (1.82 ng/g in sludge, non-detected in compost). Macrolides exhibited notable variability: azithromycin was highly concentrated in dehydrated sludge (390 ng/g) but below quantification limits (not detected) in compost, whereas clarithromycin decreased sharply from effluent (252 ng/L) to compost (0.15 ng/g).

Sulfonamides, such as sulfamethoxazole (22–46 ng/L in water, 13 ng/g in sludge) and sulfadiazine (257 ng/L in effluent), were prevalent in aqueous phases but largely undetectable (<MDL) in solid matrices. Fluoroquinolones, including ciprofloxacin and enrofloxacin, were predominantly below detection limits (<MDL) across all samples, except for ofloxacin (18 ng/g in sludge). Beta-lactams (e.g., amoxicillin, penicillins) were consistently undetectable (<MDL). Trimethoprim persisted in both water (68–116 ng/L) and sludge/compost (0.15–0.21 ng/g), indicating partial removal during treatment.

### 3.3 Physico-chemical properties of sludge

Analyzed physicochemical properties of the different sludge matrices showed a 85% of dried weight and the composted sludge 85%. Total organic matter content decreased from 53 to 38% for composted sludge due to organic matter dissipation. The pH was slightly alkaline, 7.5, in both matrices, and the C/N ratio remained stable, 7.1, indicating compost maturity. Total nitrogen was higher in dehydrated sludge (5.3–3.1%), while ammoniacal nitrogen decreased from 2.3 to 0.5% after composting, indicating volatilization and nitrification. Phosphorus levels were elevated in both sludges (92–82 mg/kg), whereas potassium, calcium, and magnesium were lower in dehydrated sludge, consistent with organic matter loss during composting which does not affect mineral compounds.

### 3.4 Heavy metal concentrations in sludge

Both sludges complied with Spanish agronomic limits (Royal Decree 1310/1990, transposing EU Directive 86/278/EEC): cadmium (5.87–4.5 mg/kg), copper (314–272 mg/kg), nickel (33–87 mg/kg), lead (81–74 mg/kg), and zinc (866–719 mg/kg) were below regulatory thresholds. Mercury in dehydrated sludge (0.28 mg/kg) slightly exceeded the composted sludge limit (<0.20 mg/kg) but remained under the maximum permissible level (10 mg/kg). Chromium (122–189 mg/kg) and arsenic (10.7–<10 mg/kg) were within safe ranges, and hexavalent chromium (<2 mg/kg) met the 2 mg/kg limit.

### 3.5 Microbiological parameters in composted sludge

*Salmonella* was not detected, and *E. coli* counts (630 CFU/g) were below the 1,000 CFU/g determined by Spanish legislation (Royal Decree 506/2013 of 28 June on fertilizer products, 2013).

## 4 Discussion

### 4.1 Antibiotics across wastewater and sludge treatments

This study provides a description of antibiotic residues and ARGs distributions along the wastewater treatment and sludge composting in a municipal WWTP in southern Spain. Across wastewater and sludge matrices, antibiotics belonging to frequently prescribed classes, particularly tetracyclines and macrolides, were consistently detected. Their occurrence aligns with consumption patterns reported in Spain (PRAN 2022–2024; Spanish Agency of Medicines and Medical Devices, AEMPS, 2022) and with previous European studies describing the persistence of these compounds in wastewater-derived environments. Due to their hydrophobic nature and relatively slow degradation rates, tetracyclines and macrolides are widely reported to accumulate in sludge, which acts as a major sink for antibiotics during treatment processes (Zhang et al., 2019). The detection of doxycycline and azithromycin in dehydrated sludge at concentrations comparable to those reported in other European and Scandinavian WWTPs further supports their classification as environmentally persistent compounds (Lindberg et al., 2005; Silvester et al., 2025).

In contrast, other antibiotic classes displayed different fate patterns. Sulfonamides and trimethoprim persisted through treatment and were detected in effluent, consistent with previous reports of partial removal in European WWTPs (O'Flynn et al., 2021), whereas *β*-lactams were not detected, in line with their known chemical instability and rapid hydrolysis rather than reduced usage (Längin et al., 2009). Together, these observations underscore that antibiotic behavior in WWTPs is compound-specific and driven by multiple interacting physicochemical and biological factors.

Notably, certain antibiotics exhibited higher concentrations in effluent than influent. Such patterns have been previously attributed to treatment-related processes, including desorption from solids, sludge remobilization, and deconjugation of metabolites during biological treatment, rather than net antibiotic production within the system (Gulkowska et al., 2008). Macrolides, in particular, may undergo reconversion from conjugated forms under alkaline conditions during secondary treatment, contributing to elevated effluent levels (Fohner et al., 2017).

### 4.2 Relationship between resistance gene profiles and the presence of antibiotic and non-antibiotic stress factors

The joint evaluation of antibiotic residues, resistance gene abundances, and non-antibiotic stressors revealed patterns of co-occurrence across wastewater and sludge matrices. These observations are interpreted descriptively and aim to contextualize potential associations rather than establish statistically supported or causal relationships, particularly given the limited number of samples.

Several ARG groups displayed parallel trends with corresponding antibiotic classes. Notably, sulfonamide resistance genes showed strong directional co-variation with sulfamethazine across matrices, a pattern consistent with previous observations in environmental and clinical settings where resistant strains persist in the presence of corresponding compounds (Andersson and Hughes, 2014). Similarly, tetracycline resistance genes exhibited positive associations with specific tetracycline derivatives, consistent with the well-described cross-resistance mechanisms encoded by ribosomal protection and efflux genes within this antibiotic class (Roberts, 2011). These patterns are best interpreted as indicative of concurrent presence rather than evidence of ongoing enrichment, selection, or horizontal gene transfer (HGT) (Gillings, 2015).

In contrast, other antibiotic classes exhibited heterogeneous or weak association patterns with their corresponding ARGs group. For example, sulfonamide resistance genes group showed variable trends with different sulfonamide compounds, and macrolide–lincosamide–streptogramin (MLSB) antibiotics displayed compound-specific associations with *erm* and *mph* genes. Associated Quinolone resistance genes also showed inconsistent patterns across individual antibiotic compounds, reflecting the known diversity of resistance mechanisms within this antibiotic class, including efflux systems and chromosomal mutations, in addition to plasmid-mediated *qnr* genes (Jacoby et al., 2014). These observations suggest that we should be prudent when drawing general conclusions.

The persistence of ARGs in matrices where corresponding antibiotics were absent or below detection limits further emphasizes the importance of historical exposure and genetic stabilization. The detection of quinolone resistance genes in the absence of measurable fluoroquinolone concentrations is consistent with previously described legacy effects, whereby resistance determinants persist long after selective pressures have diminished, particularly when associated with MGEs such as class 1 integrons (Bengtsson-Palme et al., 2018).

It is well-known that heavy metals are capable to select indirectly for antibiotic resistance by co-selection (Koditschek and Guyre, 1974) This indirect selection process is due to a coupling of the resistance mechanisms against antibiotics and heavy metals (Seiler and Berendonk, 2012; Wales and Davies, 2015). Indeed, this coselection is considered as a factor contributing to stress responses that may enhance the persistence of a shared MGEs (Pal et al., 2017; Sharma et al., 2025). In this sense, Seiler and Berendonk (2012) defined a minimum co-selective heavy metal concentration referring to dry weight of manure, and based on these reported results the concentrations studied here would allow for the occurrence of metal-driven co-selection.

### 4.3 Mobile genetic elements and sludge treatment: persistence and mitigation limits

MGEs are widely recognized as key drivers of long-term persistence of environmental resistomes. In the present study, integrons, insertion sequences and transposons commonly associated with ARGs were concurrently detected in the investigated sludge matrices, highlighting a genetic background compatible with resistance maintenance evidencing active horizontal gene transfer (HGT).

Class 1 integrons, particularly *intI1*, were among the most detected MGEs in dehydrated and composted sludges. This observation are consistent with the reported evidence of integrons as stable and ubiquitous genetic platforms in wastewater-impacted environments (Martínez et al., 2014; Gillings et al., 2015). Integrons are known to capture, store and reshuffle resistance gene cassettes, including aminoglycoside- and trimethoprim-associated determinants such as *aadA* and *dfrA*, thereby providing a mechanistic explanation for ARG persistence under a wide range of environmental conditions. Their detection supports the idea of sludge as a long-term reservoir of resistance-associated genetic material rather than as an environment characterized by active dissemination or enrichment.

In addition to integrons, insertion sequences and transposons frequently reported to co-localize with ARG cassettes were also detected. Elements such as *IS26*, *IS1247* and Tn-family transposons have been widely described in association with class 1 integrons and resistance loci in Gram-negative bacteria, enhancing the potential for genetic rearrangement and stabilization (Deng et al., 2015; Gillings et al., 2015). Previous studies have shown that genes such as *aadA* and *dfrA* are often embedded within these composite structures, particularly in environments with historical aminoglycoside or trimethoprim exposure (Shin et al., 2023).

Sludge composting substantially reduced total ARG abundance, in agreement with previous studies describing composting as an effective treatment for mitigating microbial loads and resistance determinants in organic waste (Li et al., 2024). However, the continued detection of MGEs after composting highlights important limitations of this process. Similar persistence of integrons, insertion sequences and transposons during composting has been reported elsewhere, particularly during the maturation phase, suggesting that the structural stability of MGEs exceeds that of their bacterial hosts (Zhou and Yao, 2020; Wang et al., 2024). These findings indicate that composting reduces ARG abundance but does not fully eliminate resistance-associated genetic frameworks.

Multiple, non-exclusive mechanisms may contribute to MGE persistence during sludge composting. While thermophilic phases are known to reduce microbial populations and partially degrade residual antibiotics, subsequent cooling and maturation stages may allow the survival of spore-forming or biofilm-associated bacteria, as well as the persistence of extracellular DNA adsorbed to organic matter (Liao et al., 2018; Flores-Vargas et al., 2021). Such processes also support the interpretation that residual genetic material can persist in composted sludge without necessarily reflecting ongoing HGT activity, as also proposed by Qiu et al. (2022).

Residual antibiotics and non-antibiotic stressors detected in sludge may further contribute to genetic stabilization. Experimental evidence has demonstrated that low-level stressors, including potentially toxic elements and other contaminants, can induce bacterial stress responses compatible with MGE maintenance and rearrangement (Pal et al., 2017; Wang et al., 2019; Wang et al., 2024). For instance, genes such as *bacA* have been reported to co-localize with metal resistance loci, and integrons are known to harbor both metal and antibiotic resistance determinants (Tan et al., 2023; Zhang et al., 2019).

These observations indicate that sludge composting plays an important role in reducing ARG but does not completely eliminate MGEs. This highlights the importance of considering MGEs markers in risk assessments for composted sludge application in agricultural soils. Advanced treatment strategies, such as hyperthermophilic composting, have been proposed as promising options to further reduce MGEs and residual antibiotics in sewage sludge (Pan et al., 2024).

### 4.4 ARG abundances in wastewater and sludge: ARGI and SGRI resistance indices

Resistance gene indices were used to understand ARGI profiles in wastewater and sludge, allowing for comparisons with broader European data.

**Figure 3:**
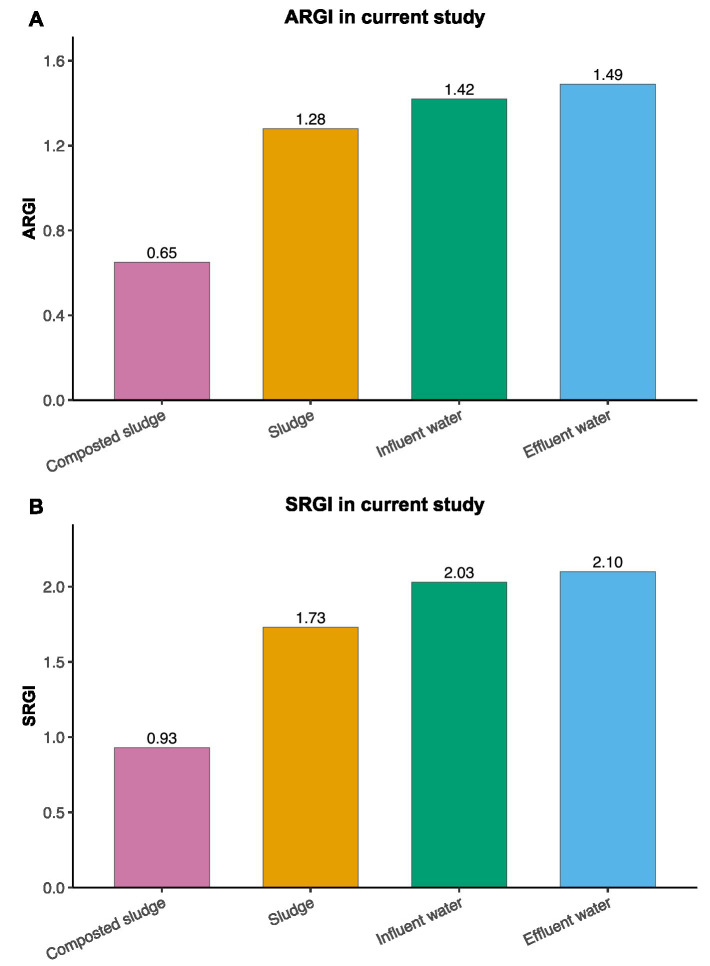
ARGI and SRGI values in current-study samples. **(A)** Antibiotic resistance gene index (ARGI) values for all sample types analyzed in the current study, including composted sludge, sludge, influent water, and effluent water. **(B)** Standardized resistance gene index (SRGI) values for the same current-study sample types. Values above bars indicate index values.

A similar Antibiotic Resistance Gene Index (ARGI) values was found between influent and effluent wastewater samples, with ARGI value of 1.42 for influent and 1.49 for effluent wastewaters (3). In contrast, sludge samples showed a clear decreasing in ARGI values after composting, diminishing from 1.248 to 0.646, before and after composting, respectively (Figure 3). This decrease is aligned with microbial community shift during the thermophilic and maturation stages of composting.

**Figure 4:**
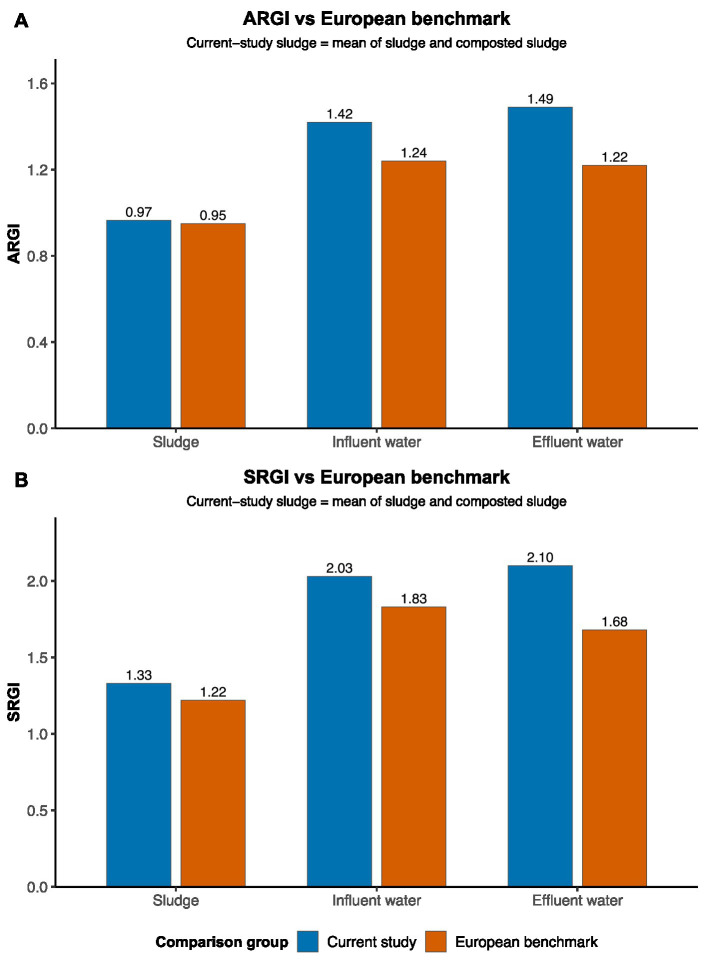
Comparison of current-study ARGI and SRGI values with European benchmark values. **(A)** Antibiotic resistance gene index (ARGI) values for sludge, influent water, and effluent water from the current study compared with corresponding European benchmark values derived from the Resistomap database. **(B)** Standardized resistance gene index (SRGI) values for the same sample types compared with the corresponding European benchmark values. For the current-study sludge category, the value shown represents the mean of the sludge and composted sludge samples. Values above bars indicate index values.

To provide a broader context for our findings, ARGI values were compared with the European data from the Resistomap database (Figure 4). The influent and effluent wastewaters ARGI values calculated from the studied WWTP fall within the European data reported averages (approximately 1.24 for influent and 1.22 for effluent wastewaters). Also, sludge samples ARGI values in this study (mean ARGI of 0.97) matched the European sludge averages (approximately 0.95), including our samples within broader regional trends.

A Standard Resistance Gene Index (SRGI) was also calculated using 62 clinically relevant ARGs. As shown in Figure 4, SRGI values exceed ARGI values, although following similar trends. The influent and effluent wastewater samples showed SRGI values of 2.03 and 2.10, respectively, which would be above European SRGI (1.83 for influent and 1.68 for effluent).

Both, ARGI and SRGI provides complementary insights into ARG distributions. ARGI reflects broader resistome patterns across matrices, while SRGI allows for standardized comparisons across studies and regions. The alignment of both indices with European reference values supports the view that the studied WWTP represents typical regional resistance profiles.

Resistance gene indices are useful tools for placing site-specific findings within larger surveillance frameworks. However, their interpretation should consider normalization effects, the composition of selected gene, and microbial community dynamics. When combined with qualitative analyses of antibiotics, ARG distributions, and MGEs, these indices contribute to a detailed understanding of antimicrobial resistance in wastewater and sludge systems, without overstating their causal or predictive implications.

### 4.5 Environmental and one health implications of ARG persistence, MGEs and clinically relevant bacterial strains

The co-occurrence of ARGs, MGEs and bacterial strains commonly associated with human diseases underscores the complexity of environmental resistomes in wastewater and sludge systems. Such environments are widely recognized as convergence zones where resistance determinants originating from clinical, agricultural, and community sources may accumulate and persist (Rizzo et al., 2013).

In the present study, bacterial groups frequently reported as environmental reservoirs of resistance determinants, including Bacteroidetes, Firmicutes and Proteobacteria, were detected alongside ARGs and MGEs across wastewater, sludge and composted matrices. These taxa have been previously shown to harbor resistance genes such as *sul1*, *ermF* and *tetM*, often embedded within integrons or transposons, providing a genetic context compatible with long-term persistence rather than acute selection (Ahmad et al., 2023; Li and Zhang, 2023). The stability of MGEs within dense and diverse microbial communities commonly found in wastewater further supports the reservoir concept.

Although opportunistic pathogens such as *Escherichia coli* and *Acinetobacter baumannii* were detected at low abundances, their presence alongside ARGs and MGEs is something that deserves attention from a One Health perspective. For instance, environmental isolates of *E. coli* have previously been reported to carry clinically relevant resistance genes, including extended-spectrum *β*-lactamases linked to insertion sequences and transferable plasmids (Gomi et al., 2022). Similarly, *A. baumannii* is well known for its capacity to acquire and regulate resistance genes such as *bla*OXA through specific insertion sequences, a mechanism described in both clinical and environmental contexts (Nguyen et al., 2020). In this study, the detection of *bla*OXA and associated insertion sequences in sludge matrices is discussed as evidence of shared genetic reservoirs.

Beyond classical ARGs, additional markers associated with tolerance, co-resistance and stress adaptation were detected across matrices, illustrating the multifactorial nature of environmental resistomes. Genes such as *qacEΔ1*, encoding tolerance to quaternary ammonium compounds, and *merA*, involved in mercury detoxification, are frequently embedded within integrons and plasmids that also carry ARGs, supporting the concept of co-localization of resistance and stress-response determinants (Mohapatra et al., 2022; Wu et al., 2023). Likewise, the detection of genes associated with resistance to rifamycins (*arr2/arr3*), nitroimidazoles (*nimE*), antimicrobial peptides (*bacA*), and streptothricins (*sat4*) reflects the broad resistome composition commonly described in wastewater-impacted environments (Thomas and Gwenin, 2021; Li et al., 2023).

In this context, the detection of persistent antibiotics of high clinical relevance further reinforces the environmental importance of antimicrobial resistance. Tigecycline, a last-resort antibiotic reserved for the treatment of multidrug-resistant infections, was detected in sludge samples (Figure 3), what highlights the potential of sludge to act as a reservoir of clinically relevant antibiotics. Similar considerations can be reached for fluoroquinolones antibiotics, which, despite being detected at trace levels, are classified as critically important antibiotics (Manaia, 2017; Meradji et al., 2025).

Likewise, the presence of the plasmid-mediated colistin resistance gene *mcr-1* in sludge is particularly noteworthy given its clinical relevance as a last-resort antibiotic resistance determinant. While its detection is reported here and does not imply active environmental dissemination, similar findings in wastewater and agricultural systems globally have raised concerns regarding the environmental dimension of critically important resistance genes (Elbediwi et al., 2019). Such observations reinforce the importance of surveillance-based approaches that monitor high-risk determinants without overstating causal implications.

The detection of phage-related gene markers, such as crAss-like bacteriophages, alongside ARGs and MGEs highlights a potential risk related with alternative pathways for increasing the environmental resistome. Indeed, bacteriophages have been previously proposed as vectors influencing on genetic pools in wastewater systems, either through transduction or by modifying microbial community dynamics (Calero-Cáceres et al., 2019).

Based on previously commented, the presence of ARGs, MGEs, opportunistic pathogens and stressors response genes in WWTPs highlights the importance of reducing selective pressures, such as excessive disinfectant use, heavy metal and toxic emerging compounds contamination, and of improving monitoring strategies for high-risk resistant determinants, such as *mcr-1*.

## 5 Conclusion

The results indicate that wastewater and sludge after being treated can act as environmental reservoirs of antimicrobial resistance. Although conventional biological treatments were effective in achieving a reduction in human pathogen bacterial strains, antibiotic residues and ARGs, other resistance determinants persisted along the different treatments. It is important to emphasize that MGEs (integrons, insertion sequences, and transposons), as well as clinically relevant genes, such as *mcr-1*, remained in wastewater and sludge matrices. This persistence could be related with a continued exposition to residual antibiotics, potentially toxic elements, and other co-occurring stressors. On the other side, ARGI and SRGI indices helped contextualize the obtained data in the present study, allowing normalization and comparison, and indicating that ARG levels are aligned with other regional previously reported analysis. In conclusion, these findings highlight the importance of monitoring for risk assessing of treated wastewater and sludge intended for agricultural use within a One Health framework, and highlight the need to bring together molecular surveillance, emerging contaminant monitoring, and resistance indices to improve the mitigation of antimicrobial resistance spread in the environment.

## Data Availability

The data presented in the study are deposited in the DIGITAL.CSIC repository using the following links to access: http://hdl.handle.net/10261/434223; http://hdl.handle.net/10261/434218.
